# Association of Antiparietal Cell and Anti-Intrinsic Factor Antibodies With Risk of Gastric Cancer

**DOI:** 10.1001/jamaoncol.2021.5395

**Published:** 2021-12-16

**Authors:** Minkyo Song, M. Constanza Camargo, Hormuzd A. Katki, Stephanie J. Weinstein, Satu Männistö, Demetrius Albanes, Heljä-Marja Surcel, Charles S. Rabkin

**Affiliations:** 1 Division of Cancer Epidemiology and Genetics, National Cancer Institute, National Institutes of Health, Bethesda, Maryland; 2 Department of Public Health Solutions, Finnish Institute for Health and Welfare, Helsinki, Finland; 3 Faculty of Medicine, University of Oulu, Oulu, Finland; 4 Biobank Borealis of Northern Finland, Oulu University Hospital, Oulu, Finland

## Abstract

**Importance:**

Autoimmune gastritis is an alternative cause of gastric carcinogenesis. This cause may be gaining importance with declining prevalence of chronic *Helicobacter pylori* infection.

**Objective:**

To determine the association of prediagnostic autoantibodies to gastric mucosa with gastric cancer (GC) risk.

**Design, Setting, and Participants:**

This cohort study used nested GC case-control analyses within separate Finnish cohorts of women of reproductive age (Finnish Maternity Cohort [FMC]; born 1938-1989) and older men (Alpha-Tocopherol, Beta-Carotene Cancer Prevention [ATBC] Study; born 1916-1939). There were 529 and 457 matched pairs from the FMC and ATBC Study, respectively, with mean participant ages of 30.5 and 57.5 years and medians of 17 and 11 years from baseline to cancer diagnosis. Data analyses were performed between August 2019 and November 2020.

**Exposures:**

Antiparietal cell antibodies (APCAs), anti-intrinsic factor antibodies, and anti–*H pylori* antibodies were measured in baseline serum using immunoassays.

**Main Outcomes and Measures:**

Autoantibody associations were estimated by odds ratios (ORs) and 95% CIs.

**Results:**

Of the 529 control participants in the FMC and 457 control participants in the ATBC Study, 53 (10%) women and 35 (7.7%) men were APCA seropositive, respectively, whereas 146 (28%) women and 329 (72%) men were *H pylori* seropositive. In the FMC, APCA seropositivity was statistically significantly associated with GC risk among *H pylori*-seronegative women (OR, 5.52; 95% CI, 3.16-9.64) but not *H pylori*-seropositive women (OR, 1.29; 95% CI, 0.64-2.60; *P* for interaction = .002). The APCA association with *H pylori* seronegativity was strongest for tumors in the fundus and corpus (OR, 24.84; 95% CI, 8.49-72.72). In the ATBC Study, APCA seropositivity was not associated with GC among either *H pylori–*seronegative men (OR, 0.99; 95% CI, 0.32-3.04) or *H pylori–*seropositive men (OR, 1.06; 95% CI, 0.60-1.88). In both cohorts, anti-intrinsic factor antibody seroprevalence was less than 2% among cases as well as controls and not statistically associated with GC risk.

**Conclusions and Relevance:**

Results of this cohort study demonstrate that autoantibody positivity may reflect subclinical autoimmune gastritis in younger women. The findings among young females and corpus subsite align with increasing cancer incidence trends for these groups. Stronger autoimmune associations in *H pylori-*seronegative individuals support a model of autoimmune gastritis replacing *H pylori* as the driving factor.

## Key Points

**Question:**

Is there an association between autoantibodies to gastric mucosa and gastric cancer (GC)?

**Findings:**

In this cohort study of 529 female matched pairs from the Finnish Maternity Cohort and 457 male matched pairs from the Alpha-Tocopherol, Beta-Carotene Cancer Prevention (ATBC) Study, prediagnostic seropositivity to antiparietal cell antibodies was associated with the elevated risk of GC among young women born in 1938 through 1989 during a median of 17 years follow-up but not among older men born in 1916 through 1939 during a median of 11 years follow-up. The magnitude of association was stronger in *Helicobacter pylori*–seronegative women and most pronounced for tumors in the corpus.

**Meaning:**

With the waning prevalence of *H pylori*, autoimmune-driven GC may explain the recent rise of GC incidence among the younger female population.

## Teaser

This cohort study seeks to determine the association of prediagnostic autoantibodies to gastric mucosa with gastric cancer risk.

## Introduction

Despite decreasing incidence over the past 50 years, gastric cancer (GC) still ranks as the fifth most common cancer and the fourth leading cause of cancer mortality worldwide.^1^ *Helicobacter pylori* is the primary risk factor for GC, and its prevalence has been declining.^2^ However, recent studies indicate that the epidemiology of GC is changing.^3,4^ Among US non-Hispanic White individuals younger than 50 years, noncardia GC increased 1.3% per year (1995-2013) despite an overall decrease in older age groups.^5^ The rising rates were stronger in counties with less than 20% prevalence of poverty, suggesting that the trends may be driven by factors other than *H pylori*, which is less prevalent among affluent individuals. The largest increase was observed for gastric corpus tumors in women. The corpus is the anatomical location where autoimmune-type gastritis is dominant.^6^ These observations—female sex, affluent population, corpus-dominant features—invite speculation that increasing prevalence of autoimmune gastritis (AIG) may be a plausible explanation for these unfavorable cancer trends.

Autoimmune gastritis is a chronic progressive inflammatory disease that replaces gastric parietal cells by atrophic and metaplastic mucosa.^7^ The destruction of parietal cells leads to reduced or absent production of acid and intrinsic factor. The resulting malabsorption and consequent vitamin B_12_ deficiency manifests clinically as pernicious anemia (PA), which is associated with GC.^8^ Patients with AIG are often asymptomatic, and when presenting with anemia they may be underdiagnosed or misdiagnosed owing to insufficient endoscopic biopsies and/or failure to explore for the underlying cause of anemia.^7^

The disappearance of ancestral constituents of the microbiota owing to improved hygiene, antibiotic pressure, and smaller family sizes is a hypothesized contributor to contemporary increases in certain autoimmune and allergic conditions.^2^ Similarly, incidence of AIG might also be on the rise. Notably, prevalence of atrophic corpus gastritis in Sweden among individuals aged 35 to 44 years nearly tripled between 1990 and 2009.^9^

Diagnosis of AIG is supported by the detection of gastric autoantibodies. Antiparietal cell antibodies (APCAs) are antibodies against gastric proton pump and are detected in approximately 90% of patients with PA.^10^ Anti-intrinsic factor antibodies (AIFAs) target proteins necessary for vitamin B_12_ absorption and are present in approximately 80% of patients with PA.^11^ For detecting atrophic gastritis, AIFAs are more specific than APCAs (100% vs 90%), but sensitivity is low (37% vs 81%),^12^ rising during disease progression.^13^ In this cohort study, we hypothesized that presence of these autoantibodies may represent asymptomatic AIG and evaluated them as potential predictive markers for GC risk.

## Methods

### Study Population

Participants with GC and controls were identified from 2 prospective population-based cohorts. The Finnish Maternity Cohort (FMC) was established in 1983 by the National Public Health Institute of Finland based on prenatal blood samples collected to screen for congenital infections and rubella immunity.^14^ This collection comprises 2 million first-trimester serum samples from 1 million women, covering approximately 94% of all Finnish pregnant women.^15^ Samples of 2 mL of serum were stored as 1 aliquot at −25 °C in a single storage place. Incident cancers were identified through record linkage with the nationwide Finish Cancer Registry through December 2014.

The Alpha-Tocopherol, Beta-Carotene Cancer Prevention (ATBC) Study was designed as a randomized prevention trial of vitamin supplementation for primary prevention of cancers in male smokers aged 50 to 69 years who were recruited from 1985 through 1988 in Finland.^16^ Fasting blood samples were collected at baseline and stored in serum aliquots at −70 °C until testing. Participants were followed up for cancer incidence using the Finnish Cancer Registry through December 2016. Data analyses were performed between August 2019 and November 2020.

Diagnoses of GC were classified according to the *International Classification of Diseases, Ninth Revision (ICD-9) *(ATBC) or *International Statistical Classification of Diseases and Related Health Problems, Tenth Revision (ICD-10)* (FMC), grouped by anatomical subsites as cardia (*ICD-9*, 151.0; *ICD-10*, C16.0); fundus and corpus (*ICD-9*, 151.1-151.2; *ICD-10*, C16.1-C16.2); antrum, pylorus, lesser curvature, and greater curvature (*ICD-9*, 151.3-151.6; *ICD-10*, C16.3-C16.6); and overlapping and unspecified (*ICD-9*, 151.8-151.9; *ICD-10*, C16.8-C16.9). Finnish law does not allow registration of race or ethnicity. According to official national statistics, 93% of Finland’s population are of Finnish and/or Swedish ancestry.^17^ All participants provided written informed consent. The FMC has a separate legal basis for the collection and scientific use by the law of the National Institute for Health and Welfare (668/2008). The original ATBC Study was approved by the institutional review boards of both the National Cancer Institute in Bethesda, Maryland, and the National Public Health Institute in Helsinki, Finland.

### Case-Control Selection

In the FMC, a total of 527 women with GC had baseline serum samples available. For each case, 1 control who had remained alive and free of GC for an equal or greater length of follow-up was randomly selected, matched for age at serum sampling (±2 years) and length of serum storage (±2 months). If a woman had more than 1 available sample preceding the index diagnosis, the earliest pregnancy sample was selected for the study. To assess longitudinal change in autoantibody levels, a serial sample from subsequent pregnancies collected more than 8 years after the first sample was selected for 35 women with GC and 35 controls. The median (range) interval between collection of the 2 samples was 10 (8-23) years.

In the ATBC Study, 457 men with GC who had available baseline serum samples were selected and matched for 1 control per case using incidence density sampling. Randomly selected controls were matched to men with GC by age at randomization (±1 year) and serum draw date (±30 days).

### Laboratory Methods

The IgG APCAs and AIFAs were analyzed using commercially available enzyme-linked immunosorbent assay (ELISA) kits (IBL International GmbH). Anti–*H pylori* whole-cell IgG antibody was tested by ELISA kits from IBL International GmbH for FMC samples and from Biohit Healthcare for ATBC Study samples. Both of these assays have comparable high sensitivity and specificity.^18^ For IBL International GmbH products, qualitative results were determined from the cutoff index calculated as the optical density of the sample divided by the mean optical density of the cutoff standard, with a cutoff index of 1 or greater considered seropositive. Quantitative autoantibody levels were estimated using a standard curve established with 6 standard calibrator samples of known concentration (U/mL) using log-linear coordinates and 4-parameter fit. Matched case-control samples and longitudinal samples of a given individual were placed in the same plate. All laboratory analyses were conducted at the University of Oulu in Finland and blinded to case-control status.

### Quality Control

To assess reproducibility, a subset of samples from the first run of each study set was retested for APCAs (FMC, 143; ATBC, 97) and AIFAs (FMC, 49; ATBC, 21). Samples were selected based on a random 50% of those with initial values that were greater than 1.2 times the cutoff, an equal number of samples that were less than 0.8 times the cutoff, and all samples that were between 0.8 and 1.2 times the cutoff. For APCAs, the resulting coefficients of variation were 6% and 7%, and intraclass correlations were 98% and 92% based on FMC and ATBC Study samples, respectively, whereas the corresponding numbers for AIFAs were 11%, 8%, 96%, and 98% respectively.

### Statistical Analysis

Differences between groups for categorical variables were assessed by χ^2^ tests. Conditional logistic regression models were used to calculate odds ratios (ORs) and 95% CIs for each antibody association with GC. Unconditional logistic regression models were used for subgroup analyses by *H pylori* serostatus and by tumor subsite. Tests for interaction were conducted using multiplicative interaction terms in the regression models. The *P *value for heterogeneity between subsites was calculated using Q-statistics. All models were adjusted for age at baseline, and ATBC models were additionally adjusted for trial supplementation group, decided a priori for a parsimonious model. Differences between quantitative antibody levels for serial samples were assessed using the paired *t* test. Population attributable risk (PAR) and 95% CIs were calculated using function AFclogit for matched case-control set analysis and AFglm for unmatched analyses from R package AF, version 0.1.5 (R Foundation).^19^ These functions estimate the attributable fraction for binary outcomes under the hypothetical scenario where a binary exposure is eliminated from the population. All other analyses were conducted using SAS, version 9.4 (SAS Inc), and statistical significance was based on 2-sided *P* < .05.

## Results

### Baseline Characteristics

Within each cohort, cases and controls were similar at the time of blood collection (eTable in the Supplement). The median (IQR) interval from blood sample to diagnosis was 17.4 (10.2-23.3) years for FMC cases and 11.0 (6.0-18.0) years for ATBC Study cases. Participants in the FMC were substantially younger than participants in the ATBC Study at time of blood collection (mean [SD] age, 30.5 [5.9] vs 57.5 [4.9] years; *P* < .001); participants with GC in the FMC were younger than in the ATBC Study at diagnosis (mean [SD] age, 47.4 [9.8] vs 69.5 [7.5] years; *P* < .001). The 2 cohorts had considerable overlap in time periods of sample collection and cancer diagnosis but represented nearly distinct birth cohorts: post-1940 vs pre-1940.

### Antibody Associations With GC

In both cohorts, baseline *H pylori* seroprevalence was higher among GC cases than controls: 394 of 529 (74.5%) vs 146 of 529 (27.6%), respectively, for the FMC, and 399 of 457 (87.3%) vs 329 of 457 (72.0%), respectively, for the ATBC Study (Table). The ORs for the association of *H pylori* seropositivity with cancer risk were 7.38 (95% CI, 5.28-10.31) in the FMC and 2.81 (95% CI, 1.95-4.06) in the ATBC Study.

**Table.** Association of APCA and AIFA Seropositivity With Gastric Cancer Overall and Stratified by *Helicobacter pylori* Serostatus

Participants	FMC				ATBC Study			
	No. (%)		OR (95% CI)^a^	*P* value for interaction^b^	No. (%)		OR (95% CI)^a^	*P* value for interaction^b^
	Cases (n = 529)	Controls (n = 529)			Cases (n = 457)	Controls (n = 457)		
**All**								
APCA positive	93 (17.6)	53 (10.0)	1.85 (1.30-2.64)	NA	33 (7.2)	35 (7.7)	0.98 (0.60-1.62)	NA
AIFA positive	10 (1.9)	6 (1.1)	1.66 (0.60-4.56)	NA	6 (1.3)	2 (0.4)	4.97 (0.56-43.83)	NA
*H pylori* positive	394 (74.5)	146 (27.6)	7.38 (5.28-10.31)	NA	399 (87.3)	329 (72.0)	2.81 (1.95-4.06)	NA
***H pylori* negative**								
APCA positive	53 (39.3)	39 (10.2)	5.52 (3.16-9.64)	.002	5 (8.6)	11 (8.6)	0.99 (0.32-3.04)	.91
AIFA positive	5 (3.7)	3 (0.8)	2.09 (0.46-9.47)	.21	0	0	NA	NA
***H pylori* positive**								
APCA positive	40 (10.2)	14 (9.6)	1.29 (0.64-2.60)	NA	28 (7.0)	24 (7.3)	1.06 (0.60-1.88)	NA
AIFA positive	5 (1.3)	3 (2.1)	0.51 (0.10-2.56)	NA	6 (1.5)	2 (0.6)	3.80 (0.41-34.92)	NA

Abbreviations: AIFA, anti-intrinsic factor antibody; APCA, antiparietal cell antibody; ATBC, Alpha-Tocopherol, Beta-Carotene Cancer Prevention; FMC, Finnish Maternity Cohort; NA, not applicable; OR, odds ratio.

^a^ ORs and 95% CIs by conditional logistic regression models for each antibody were calculated separately, adjusted for age at blood draw (both studies) and additionally for type of intervention (ATBC Study only).

^b^ Calculated for *H pylori* positive vs negative.

In the FMC, baseline APCA seropositivity was higher among GC cases (93 of 529 [17.6%]) than controls (53 of 529 [10.0%]), with an OR of 1.85 (95% CI, 1.30-2.64). Stratified by *H pylori* serostatus, the association was statistically significantly stronger in *H pylori*–seronegative women (OR, 5.52 [95% CI, 3.16-9.64]) than in *H pylori*–seropositive women (OR, 1.29 [95% CI, 0.64-2.60]; *P* = .002). However, baseline APCA seropositivity (cases, 33 of 457 [7.2%]; controls, 35 of 457 [7.7%]) was not associated with GC risk in the ATBC Study overall (OR, 0.98 [95% CI, 0.60-1.62]), nor separately among men who were *H pylori* seronegative (OR, 0.99 [95% CI, 0.32-3.04]) or *H pylori *seropositive (OR, 1.06 [95% CI, 0.60-1.88]).

In both cohorts, baseline AIFA seroprevalence was less than 2% among both GC cases and controls, and ORs for association were not statistically significant. Furthermore, there were no statistically significant AIFA associations for strata defined by *H pylori* serostatus in either cohort.

### Antibody Associations With GC Subsites by *H pylori* Seroprevalence in the FMC

For both *H pylori* serostatus strata, the strongest association was observed for cancers of the fundus and corpus, which represented 70 of 529 (13.2%) GCs (Figure). While both ORs were considerably elevated, the increase was nearly an order of magnitude greater for *H pylori*–seronegative individuals than for *H pylori*–seropositive individuals (24.84 vs 2.64; *P *for interaction = .001). The APCA association among *H pylori*–seronegative individuals with antrum, pylorus, and curvatures subsites was not statistically significant (OR, 3.27 [95% CI, 0.83-12.86]).

**Figure.**
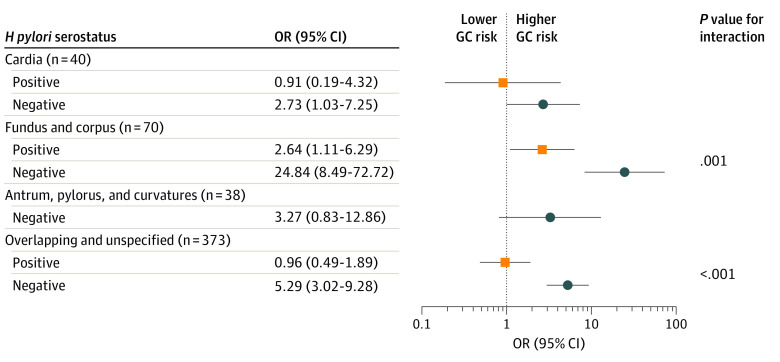
Association of Antiparietal Cell Antibodies (APCAs) With Gastric Cancer (GC) by Anatomical Subsite and *Helicobacter pylori* Serostatus Data are from the Finnish Maternity Cohort. Odds ratios (ORs) and 95% CIs are by unconditional logistic regression adjusted for age at sample. There were no APCA-seropositive individuals among *H pylori*–seropositive antrum, pylorus, and curvatures cases.

In addition, among *H pylori*–negative individuals, the APCA association with fundus and corpus was statistically significantly different from the association with antrum, pylorus, and curvatures (24.84 vs 3.27; *P *for heterogeneity = .02). In an extreme hypothetical scenario of redistributing all overlapping and nonspecified tumors to antrum, pylorus, and curvature subsites, this difference remains with an OR of 5.03 (95% CI, 2.93-8.64; *P *for heterogeneity = .01).

### Seroprevalence by Birth Cohort in the FMC

Seropositivity against *H pylori* gradually declined in successive birth cohorts (pre-1950 and post-1970) among both cases (59 of 68 [86.8%] to 32 of 44 [72.7%], respectively) and controls (29 of 70 [41.4%] to 11 of 43 [25.6%], respectively). In contrast, APCA seroprevalence increased over time among cases (11 of 68 [16.2%] to 10 of 44 [22.7%], respectively) and did not appreciably change among controls (7 of 70 [10.0%] to 4 of 43 [9.3%], respectively) (eFigure 1 in the Supplement).

### Longitudinal Change of APCAs in the FMC

Quantitative APCA levels were relatively unchanged between paired samples obtained 8 to 23 years apart, except for 3 individuals (mean, 6.2 U/mL vs 6.1 U/mL; *P* = .99; eFigure 2 in the Supplement). Two of these individuals were seropositive cases who nominally seroreverted with levels near the cutoff at both time points, while 1 was a seroconverting control with a large magnitude increase.

### Population-Attributable Fraction of APCAs in the FMC

The estimated PAR from APCAs among *H pylori*–seronegative individuals was 32.4% (95% CI, 21.6%-43.1%). This accounts for 8.1% (95% CI, 4.2%-12.0%) of GC overall.

## Discussion

In this large, prospective cohort study with a long follow-up, APCA seropositivity was associated with subsequent development of GC in women born since 1938. The strength of association was restricted to *H pylori*–seronegative women with the highest OR for fundus and corpus tumors. In the comparative cohort of older men, APCA and AIFA seropositivity were not associated with GC regardless of *H pylori* serostatus. To our knowledge, this study is the first attempt to quantify the PAR for AIG in gastric carcinogenesis.

Autoimmune gastritis is a well-recognized precursor of PA and may develop into GC, but its quantitative contribution to GC in the general population is unknown. Previous studies based on histology rather than serology had small sample sizes.^20,21,22^ Studies reporting GC associations with AIG were actually investigations of PA.^23^ Moreover, PA diagnoses were based on questionnaire or vitamin B_12 _deficiency, which invites questions about validity, including possible causes of vitamin B_12_ deficiency other than AIG.

Both APCAs and AIFAs have been used to explore the pathologic sequence leading to GC. Cross-sectional analyses found higher APCA levels associated with either histologically confirmed corpus atrophy or serologic atrophy.^24,25^ Retrospective studies have examined APCA and AIFA seropositivity in patients with PA.^26^ The few case-control studies investigating autoantibody levels with GC had controversial results. A study comparing patients with GC with healthy controls found no association of APCAs and AIFAs with GC,^27^ whereas another found higher APCA seroprevalence in patients with GC compared with healthy controls.^25^ One prospective study from China (an endemic area of cardia GC and esophageal cancer) reported an inverse association of APCAs with cardia adenocarcinoma and no statistically significant association with noncardia GC, contrary to expectation.^28^

We demonstrated an independent association of AIG with GC. The effect modification by *H pylori* status suggests existence of 2 sets of cases with differing causes. The association of APCAs with GC was most pronounced for cancers of the fundus and corpus, where targets of APCAs are mainly located.^6,7^ These 2 related observations support interpretation of APCAs as reflecting autoimmune corpus restricted–type atrophy that leads to GC development.

Lack of association in the ATBC Study may be because of the overall low prevalence of autoantibodies in this population. Like other autoimmune disorders, AIG is also known to be less prevalent in men.^29^ Cigarette smoking is inversely associated with prevalence of APCAs.^30^ Conceivably, the men in the ATBC Study, who were all smokers, may have had lower APCA prevalence than nonsmoking men. Further adjustment of the present statistical models by cigarette pack-years did not change the findings. Considering the increasing secular trends in autoimmune diseases,^9^ the later birth years of the women in the FMC may have contributed to their higher prevalence of autoantibodies as compared with the men in the ATBC Study. Furthermore, the mean age at diagnosis of cases in the FMC was lower than the cases in the ATBC Study, consistent with the relatively younger age distribution of AIG-associated GC.^31^

To our knowledge, there are no data about associated levels of APCAs and AIFAs, but other autoantibodies have been reported to change during pregnancy. Antithyroid antibodies are noted to decrease,^32^ whereas antiphospholipid antibodies increase in pregnancy.^33^ While pregnancy may alter APCAs and AIFAs, we do not have a presumption about the direction of change. Because we are matching for gestation, the mean effect of pregnancy at a given duration should be canceled out in case-control comparisons. Most baseline measurements were from first pregnancies. In follow-up pregnancies, most women had unchanged autoantibody levels.

The AIFA seroprevalence was low in both the FMC and ATBC Study. The AIFAs are known to be less sensitive and more specific in detecting AIG.^12,13^ Although the prevalence of AIFAs was too low in these healthy individuals to have enough statistical power, the direction of association was positive in both cohorts.

This study has several strengths. First, the FMC is a unique population-based cohort that covers the age and birth-year range of our interest. Most prospective cohort studies targeting chronic disease outcomes, such as cardiovascular events or cancer, enroll participants around middle age. Median age at onset of PA is 30 to 39 years old, implying onset of AIG that precedes this age range. Although we could not include a contemporaneous group of men, evaluating men from an earlier period was informative to show their contrasting absence of an autoantibody-GC association. Second, to our knowledge, this is the largest prospective study investigating the association of autoantibodies with GC risk. Third, we had serial samples from the same individuals over a long period of time to assess the change in antibody levels. Notably, the autoantibody levels did not change over time, supportive of the robustness of the present findings. Similarly, in a study of a Japanese population, long-term administration of proton pump inhibitors and *H pylori* eradication did not influence APCA levels.^24^ The 2 seropositive cases in the present study that nominally seroreverted may have lost quantitative levels owing to disease progression to severe atrophic gastritis,^34^ while the 1 control with seroconversion may have developed AIG later in life. Fourth, using a commercial ELISA for autoantibody detection with high reproducibility allows for replication in other studies.

### Limitations

Nevertheless, this study has some limitations. We did not have information on *H pylori* eradication therapy or other medication use that could influence GC risk. However, we found a strong association of *H pylori* seropositivity with GC. Autoantibody seropositivity may not necessarily imply existence of an underlying clinical disease. Autoimmune conditions frequently co-occur,^35,36,37^ and APCAs and AIFAs may be found in other autoimmune diseases or even in healthy people.^10^ It is currently unknown whether these autoantibodies have a pathophysiologic role outside of the stomach or reflect underdiagnosed AIG accompanying other autoimmune disorders. Anatomical subsite was classified as overlapping or unspecified for a large proportion of cases. Because there is no differential clinical management based on subsite, this variable should not have biased the results. Moreover, the sensitivity analysis assuming all overlapping and nonspecified cases were antrum, pylorus, and curvature tumors did not eliminate the preferential association with the fundus and corpus subsites. Furthermore, we did not have any clinical information with regard to clinical stage or histology of the cancer cases, nor were we able to assess precancerous endoscopic findings to assess the degree of gastric atrophy.

Future studies should incorporate more diverse populations and birth cohorts. While this study demonstrates association of antibodies with disease, follow-up serosurveys are needed to determine whether antibody prevalence has increased over time. Although autoantibody prevalence did not change much among controls, this study suggests a relative increasing trend of autoantibody-associated GC cases in contrast with decreasing *H pylori*–associated GC cases in later years. These complementary findings provide a clue that autoimmune-driven GC may be replacing *H pylori*–driven GCs. With decreasing *H pylori* prevalence in the general population, it seems likely that autoimmune-driven cases will determine future trends in GC incidence.

## Conclusions

Increasing trends among younger generations portend future rises in GC incidence overall. In this cohort study, we tested a novel but plausible etiologic explanation for this alarming trend. The use of prediagnostic biospecimens avoids potential disease bias that would result from a cross-sectional comparison and ensures validity of any detected associations. Wider assessment of autoantibodies in patients with gastritis or GC may uncover a hidden cause for strategies to reduce the public health burden of GC.

## Notes

Supplement. eTable 1. Selected characteristics of gastric cancer cases and controls, FMC and ATBC Study eFigure 1. Seropositivity for anti–*Helicobacter pylori* antibody and APCA by birth year, Finnish Maternity Cohort eFigure 2. Longitudinal change of quantitative APCA levels by *Helicobacter pylori* serostatus, Finnish Maternity Cohort
