# Supplementary material for: Association of Antiparietal Cell and Anti-Intrinsic Factor Antibodies With Risk of Gastric Cancer
Source: JAMA Oncol. 2021 Dec 16;8(2):1–7. doi: 10.1001/jamaoncol.2021.5395 (PMC8678897; doi:10.1001/jamaoncol.2021.5395)
Supplement: Supplement. — eTable 1. Selected characteristics of gastric cancer cases and controls, FMC and ATBC Study eFigure 1. Seropositivity for anti–Helicobacter pylori antibody and APCA by birth year, Finnish Maternity Cohort eFigure 2. Longitudinal change of quantitative APCA levels by Helicobacter pylori serostatus, Finnish Maternity Cohort [file jamaoncol-e215395-s001.pdf]

Supplemental Online Content

Song M, Camargo MC, Katki HA, et al. Association of antiparietal cell and anti-intrinsic factor antibodies with risk of gastric cancer. *JAMA Oncol*. Published online December 16, 2021. doi:10.1001/jamaoncol.2021.5395

eTable. Selected characteristics of gastric cancer cases and controls, FMC and ATBC Study

eFigure 1. Seropositivity for anti–*Helicobacter pylori* antibody and APCA by birth year, Finnish Maternity Cohort

eFigure 2. Longitudinal change of quantitative APCA levels by *Helicobacter pylori* serostatus, Finnish Maternity Cohort

This supplemental material has been provided by the authors to give readers additional information about their work.

© 2021 Song M et al. *JAMA Oncol.*

eTable. Selected characteristics of gastric cancer cases and controls, FMC and ATBC Study

	FMC						ATBC Study				
	Cases (N=529)		Controls (N=529)				Cases (N=457)		Controls (N=457)		
	n	%	n	%	p-value		n	%	n	%	p-value
Age at blood sampling, year											
16-19	21	4.0	20	3.8	0.96		-	-	-	-	0.78
20-29	232	43.9	233	44.1			-	-	-	-	
30-39	247	46.7	247	46.7			-	-	-	-	
40-49	29	5.5	29	5.5			-	-	-	-	
50-59	-	-	-	-			303	66.3	307	67.2	
60-69	-	-	-	-			154	33.7	150	32.8	
Year of blood sampling											
1980-1989	363	68.6	366	69.2	0.88		457	100	457	100	1.00
1990-1999	130	24.6	127	24.0			-	-	-	-	
2000-2009	33	6.2	33	6.2			-	-	-	-	
2010-2019	3	0.6	3	0.6			-	-	-	-	
Birth year											
1910-1919	-	-	-	-	0.97		18	3.9	10	2.2	0.72
1920-1929	-	-	-	-			208	45.5	218	47.7	
1930-1939	1	0.2	3	0.6			231	50.6	229	50.1	
1940-1949	67	12.7	67	12.7			-	-	-	-	
1950-1959	255	48.2	249	47.1			-	-	-	-	
1960-1969	162	30.6	167	31.6			-	-	-	-	
1970-1979	38	7.2	37	7.0			-	-	-	-	

© 2021 Song M et al. *JAMA Oncol.*

1980-1989	6	1.1	6	1.1			-	-	-	-	
Interval from blood sampling to diagnosis, year											
< 1	10	1.9					11	2.4			
1-9	120	22.7					199	43.5			
10-19	186	35.2					164	35.9			
≥20	213	40.3					83	18.2			
Age at diagnosis, year											
20-29	20	3.8					-	-			
30-39	95	18.0					-	-			
40-49	197	37.2					-	-			
50-59	169	32.0					43	9.4			
60-69	45	8.5					181	39.6			
70-79	3	0.6					179	39.2			
80-89	-	-					54	11.8			
Year of cancer diagnosis											
1980-1989	16	3.0					45	9.9			
1990-1999	101	19.1					213	46.6			
2000-2009	231	43.7					155	33.9			
2010-2019	181	34.2					44	9.6			

p-values calculated by Mantel-Haenszel chi-square

© 2021 Song M et al. *JAMA Oncol.*

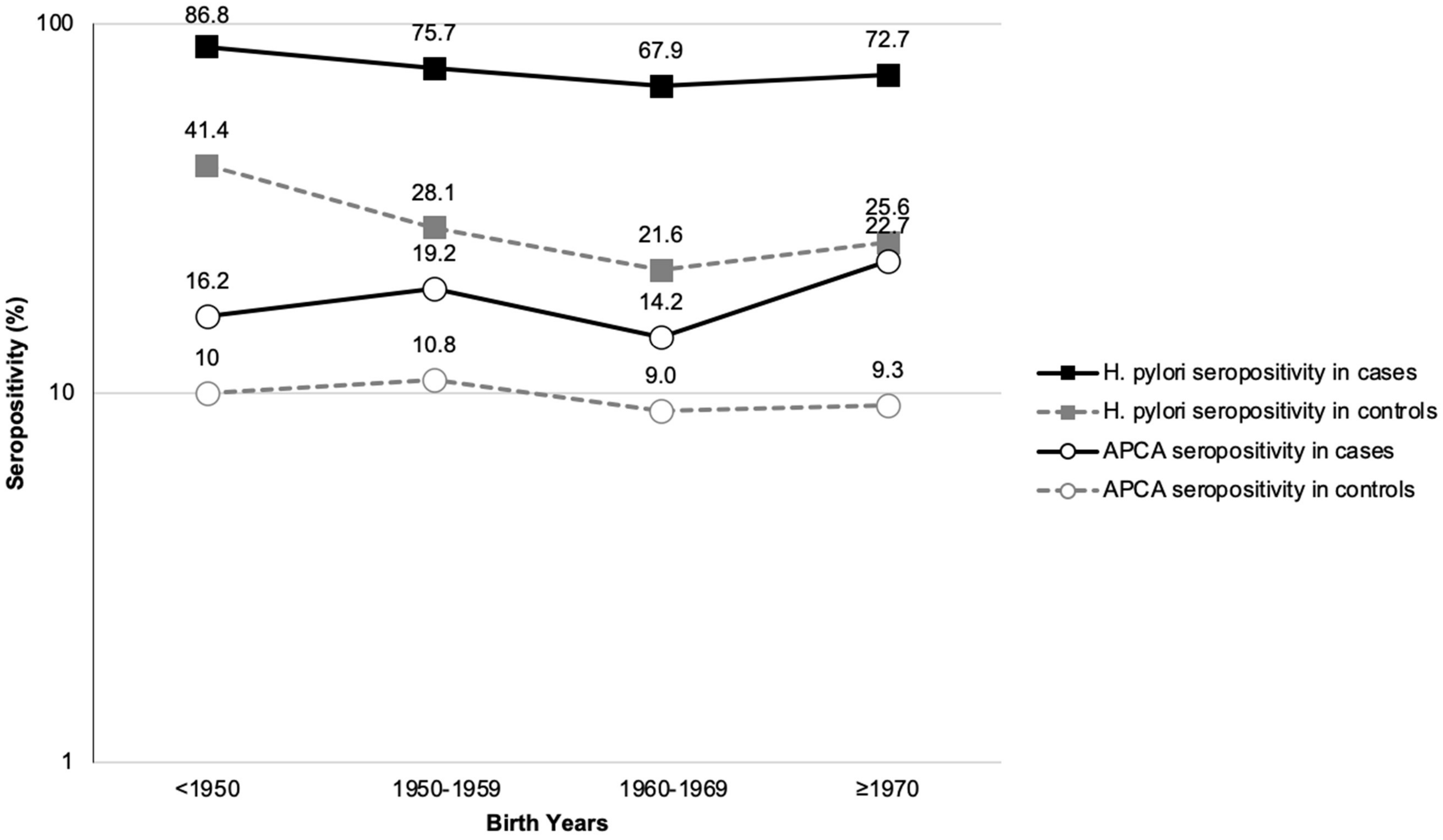

eFigure 1. Seropositivity for anti–*Helicobacter pylori* antibody and APCA by birth year, Finnish Maternity Cohort

© 2021 Song M et al. *JAMA Oncol.*

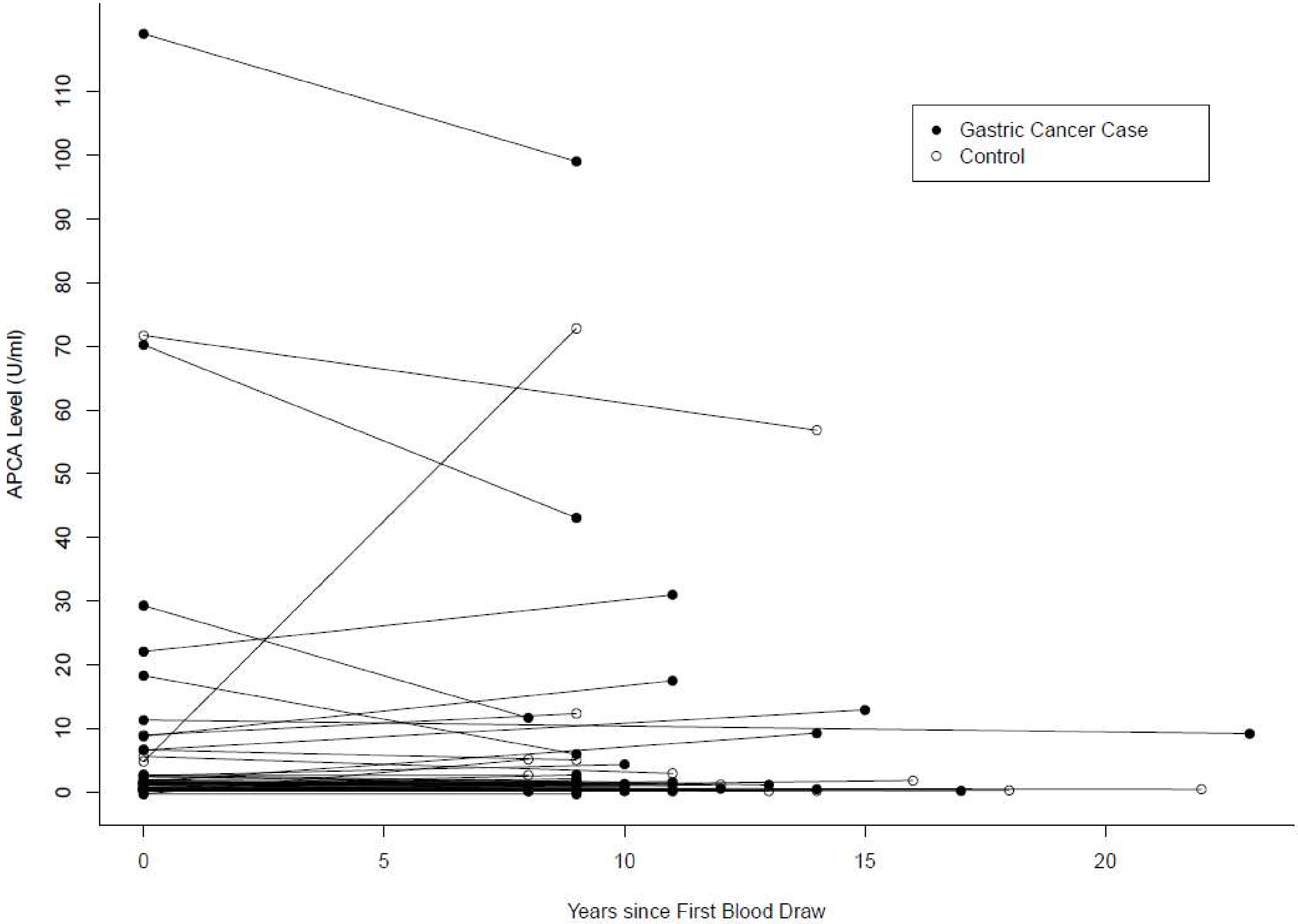

eFigure 2. Longitudinal change of quantitative APCA levels by *Helicobacter pylori* serostatus, Finnish Maternity Cohort

© 2021 Song M et al. *JAMA Oncol.*
